# A Novel Intraoperative Posture‐Adjustment Apparatus for Correction of Cervical Lordosis in Anterior Cervical Surgery

**DOI:** 10.1111/os.13917

**Published:** 2023-10-22

**Authors:** Yifei Deng, Beiyu Wang, Hao Liu, Yang Meng, Xin Rong, Tingkui Wu, Hao Chen, Ying Hong

**Affiliations:** ^1^ Department of Orthopedic Surgery, West China Hospital Sichuan University Chengdu China; ^2^ Department of Anesthesiology, West China Hospital Sichuan University Chengdu China; ^3^ Department of Operation Room, West China Hospital Sichuan University Chengdu China

**Keywords:** Anterior cervical surgery, Cervical alignment, Intraoperative, Posture‐adjustment apparatus, Kyphosis

## Abstract

**Objective:**

Cervical alignment is a crucial factor related to the success of anterior cervical surgical procedures. In patients with severe spinal cord compression, a traditional neck pillow (TNP) may not adequately correct cervical position during surgery. Therefore, the aim of this study was to introduce this innovative intraoperative posture‐adjustment apparatus (IPAA), and explored its clinical and radiological results in cervical angle correction against TNP in patients who had undergone anterior cervical surgery.

**Methods:**

The clinical and radiological data of 86 patients who underwent anterior cervical surgery with a minimum follow‐up period of 1 year were retrospectively reviewed. Of these, 58 patients underwent IPAA, whereas 28 underwent TNP. Radiological parameters such as the degree of C2‐C7 lordosis (CL), functional spinal unit angle (FSUA), C7 slope (C7S), fusion rate, and adjacent segment disease (ASD) were recorded and compared between the groups. Clinical outcomes including the Japanese Orthopaedic Association (JOA), neck disability index (NDI), visual analogue scale (VAS) for neck and arm were recorded. Complications such as kyphosis, dysphagia, Braden Scale score, revision surgery, hematoma, cerebrospinal fluid leakage, wound infection, and deep venous thrombosis were also recorded. The independent *t*‐test or Mann–Whitney U test was used to compare continuous data, and categorical variables were assessed using the Pearson's chi‐square test or Fisher's exact test.

**Results:**

Compared with the pre‐operative data, the post‐operative CL, FSUA, and C7S were significantly increased in both groups. CL, FSUA, and C7S in the IPAA group (14.44 ± 4.94°, 7.36 ± 2.91°, 16.54 ± 4.63°) were significantly higher than those in the TNP group (7.17 ± 8.19°, 4.99 ± 5.36°, 14.19 ± 4.48°; P < 0.05). Although there were no significant differences between the groups in terms of VAS arm and JOA scores, the post‐operative and final follow‐up NDI and VAS neck scores in the IPAA group were significantly lower than those in the TNP group (*p* < 0.05). At the last follow‐up, the TNP group had significantly more kyphotic patients than the IPAA group (2 *vs.* 0, *p* = 0,041). There was no significant difference between the groups in terms of fusion rate, ASD, or complications such as dysphagia, Braden's Scale score, revision surgery, hematoma, cerebrospinal fluid leakage, wound infection, or deep venous thrombosis.

**Conclusion:**

IPAA was shown to be more effective than TNP in adjusting cervical alignment (CL, FSUA, and C7S). These findings suggest that IPAA could be used as an alternative way to TNP in neck setting and cervical alignment adjustment and IPAA could potentially improve clinical outcomes after anterior cervical surgery.

## Graphical abstract

When conducting anterior cervical surgery, surgeons prefer to set the neck into a slightly extended position which could help patients to acquire an optimal cervical angle. Patients with severe spinal cord compression are not suitable for extended position to avoid spinal cord injury. So, we innovatively invent an intra‐operative posture adjust apparatus to help patients to acquire an optimal cervical angle and avoid iatrogenic spinal cord injury.

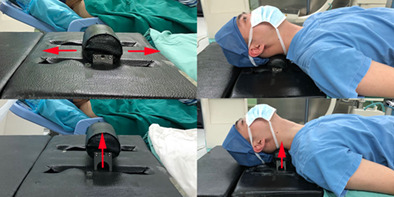

## Introduction

Cervical degenerative disc disease (CDDD) is a chronic and progressive pathology frequently presenting with neck and arm pain and occasionally accompanied by spinal dysfunction and neurological deficits. The recommended therapeutic choice for patients with CDDD who are unresponsive to regular conservative treatments is surgical treatment. Of the available surgical techniques, anterior cervical discectomy and fusion (ACDF) has become the gold standard surgical technique for decompressing the spinal cord and nerve roots over the past several decades.^1^, ^2^, ^3^, ^4^ However, with the widespread application of ACDF, related complications have gradually increased, with adjacent segment disease (ASD) being the most common. Therefore, cervical disc replacement (CDR) has been designed to lower the incidence of ASD and preserve range of motion at the surgical level.^5^, ^6^, ^7^ However, not all of these operated levels are appropriate for CDR in multilevel CDDD. To treat multilevel CDDD, hybrid surgery (HS), which incorporates ACDF and CDR, has been developed.^8^, ^9^, ^10^ All three surgical interventions (ACDF, CDR, and HS) fall within the category of anterior cervical surgery and require specialized neck posture‐establishing procedures.

**Fig. 1:**
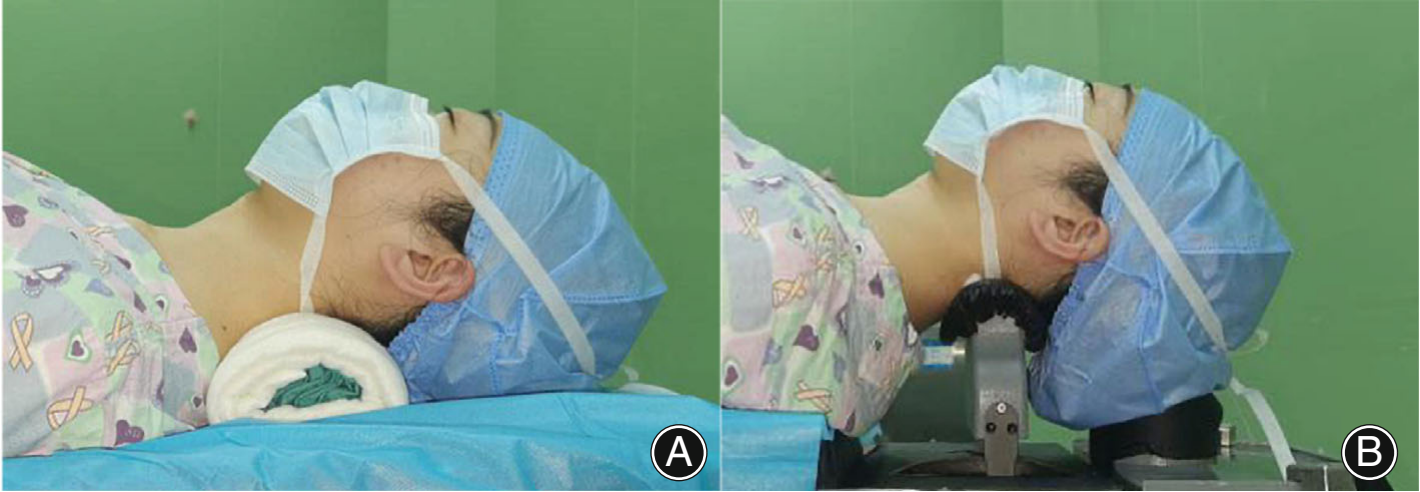
Comparison of traditional neck pillow and intraoperative posture‐adjustment apparatus.

When performing anterior cervical surgery, surgeons prefer to place the neck in a slightly extended position, as this may assist patients in obtaining an ideal cervical angle post‐surgically and allows surgeons to expose the cervical spine. This is achieved *via* the use of a traditional neck pillow (TNP) (Fig. 1A). However, the extended neck position is not always safe, especially in patients with severe spinal myelopathy. According to Hindman *et al*., cervical cord injury claims (*n* = 48) account for 0.9% of all claims for general anesthesia (*n* = 5231). Of these, cervical spine procedures are responsible for more than half (*n* = 26) of reported injuries to the cervical spinal cord. Moreover, head and neck position during cervical procedures is the predominant contributor to cervical cord injury.^11^

Neck extension position may exacerbate spinal cord injury for multiple reasons; cord compression can occur posteriorly from the thickening and buckling of the ligamentum flavum and anteriorly from disc herniation, osteophytes, or ossification of the posterior longitudinal ligament, which is aggravated by extension.^12^, ^13^, ^14^ Dynamic imaging investigations have demonstrated that cervical flexion or—more frequently—extension can significantly worsen spondylitis cord compression that was already present in the neutral position.^15^, ^16^, ^17^

To avoid iatrogenic spinal cord injury during cervical surgery, neck extension tests are often performed beforehand. According to James, in a neck extension test, the patient extends their neck to maximal extension and maintains that position for 15–25 s.^18^ Aggravation of any neurological or spinal dysfunction is defined as a positive neck extension test result. If the result is positive, the neck is placed at an approximately horizontal level to avoid iatrogenic spinal injury. Consequently, such patients may not achieve satisfactory cervical alignment owing to poor neck position. Overall, a posture‐adjustment apparatus for anterior cervical surgery remains lacking. Therefore, to better adjust the cervical angle and avoid iatrogenic spinal cord injury, we designed an innovative intraoperative posture‐adjustment apparatus (IPAA) to modify the ideal cervical angle during anterior cervical surgery (Fig. 1B). Once decompression factors such as herniated discs were removed, we intraoperatively manipulated the neck into an extended position using this apparatus.

In this article, we introduce a novel intraoperative technique for positioning the neck in an optimally extended position by applying an IPAA. With the help of the IPAA, changing the position during anterior cervical surgery could become a reality. The purpose of this study was to: (i) illustrate the effectiveness and safety of adjusting the cervical alignment using the IPAA; and (ii) compare the clinical and radiological outcomes between IPAA and TNP.

Theoretically, this device may improve cervical alignment while ensuring safety because there would be no more compression factors to the spinal cord when setting the neck to an extended position.

## Methods

### Patient Population

This retrospective, clinical, and comparative study was approved by the Ethics Committee of the West China Hospital (No. 2020‐744). All experiments were performed in accordance with the Declaration of Helsinki. Between January 2019 and December 2021, 86 consecutive patients who underwent anterior cervical surgery for ACDF, CDR, or HS using either IPAA or TNP were retrospectively examined and analyzed. A total of 58 patients who were provided an IPAA were assigned to the IPAA group, while 28 who were administered TNP were assigned to the TNP group. The average age in the IPAA group was 49.64 ± 10.08 years, and was 46.57 ± 9.73 years in the TNP group; the age difference between the two groups was not statistically significant. In total, 46 patients underwent ACDF, 13 underwent CDR, and 27 underwent hybrid ACDF. The mean follow‐up period was 14.86 ± 3.74 months for observation of the short‐term outcomes of the apparatus (detailed information is provided in Table 1).

**TABLE 1:** Summary of the patient demographic data (displayed as a number or mean ± standard deviation)

Variable	IPAA group (*n* = 58)	TNP group (*n* = 28)	*t/x* ^*2*^ */z*	*p*‐value
No. of patients, *n*	58	28		‐
Gender (M/F)	17/41	13/15	2.436	0.119
Age, year	49.64 ± 10.08	46.57 ± 9.73	1.337	0.185
Levels			2.060	0.357
One‐level	15	10		
Two‐level	22	12		
Three‐level	21	6		
Surgery			0.851	0.653
ACDF	30	16		
CDR	8	5		
Hybrid	20	7		
Follow‐up time	14.56 ± 3.61	15.48 ± 3.99	−1.051	0.297

Abbreviations: ACDF, anterior cervical discectomy and fusion; CDR, cervical disc replacement; IPAA, intraoperative posture‐adjustment apparatus; TNP, traditional neck pillow.

The inclusion criteria were as follows: (i) patients with CDDD between C3 and C7 with radiculopathy and/or myelopathy who did not respond to at least 6 weeks of conservative treatment; (ii) those with symptoms consistent with radiological materials; and (iii) those on whom ACDF, CDR, or HS was conducted on a single‐level or multi‐level. The exclusion criteria were as follows: (i) patients with history of previous cervical spine surgery; (ii) those with unavailable radiological parameters; and (iii) those with other cervical diseases such as infections, tumors, or fractures.

### Necessity for IPAA and Apparatus Details

The purpose of the IPAA is to achieve intraoperative neck adjustment and to assist patients in achieving better cervical alignment and avoiding iatrogenic spinal cord injury caused by inappropriate neck positioning. Intended to be primarily applied in anterior cervical surgery, the underlying mechanism of IPAA is that the spinal canal becomes sufficiently large enough when performing posture‐setting procedures. In other words, even in an extremely extended position, compression factors such as herniated discs, osteophytes, and posterior longitudinal ligaments are no longer forced on the spinal cord.

**Fig. 2:**
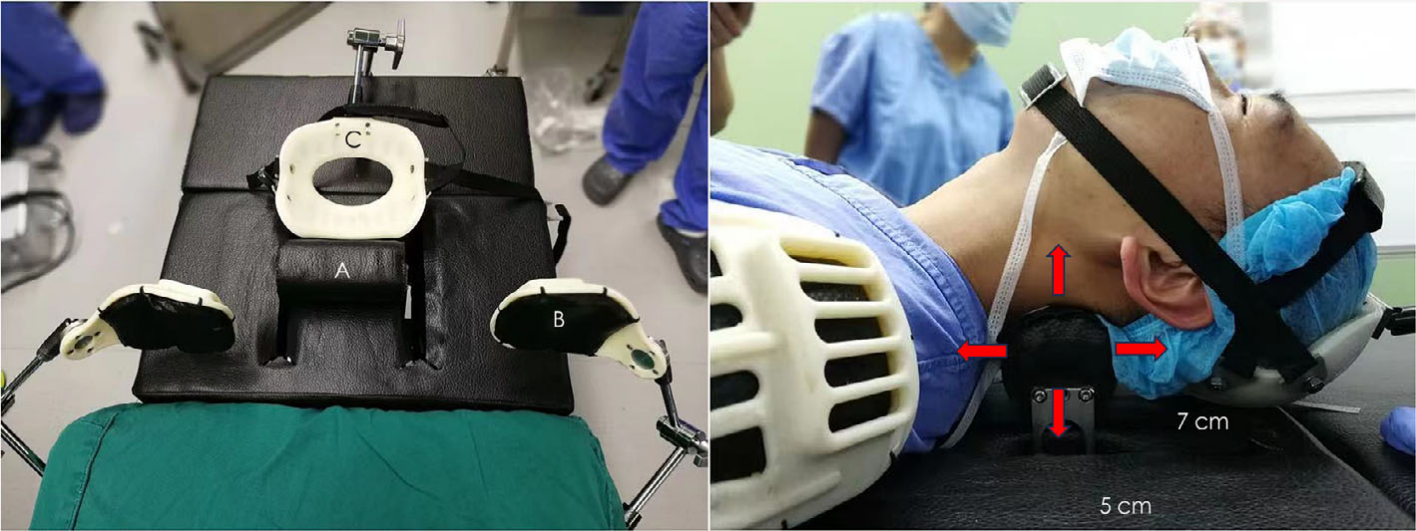
Illustration of intraoperative posture‐adjustment apparatus. A is a cervical electric pillow, which can move cranially and caudally (7 cm) and vertically to alter the curvature of the cervical spine. B represents the shoulder‐pressing part, which can press the shoulder to fully expose the lower cervical vertebra. C is head immobilization device with 30° of horizontal rotation. The red arrows represent the cervical electric pillow's moving direction.

The IPAA consists of three parts (Fig. 2): (i) a cervical electric pillow, composed of internal carbon fiber and an external polyurethane sponge, which can alter the curvature of the cervical spine through cranial and caudal (7 cm) and vertical (5 cm) movements; (ii) a shoulder‐pressing device consisting of a nylon skeleton and a polyurethane sponge that can press the shoulder to fully expose the lower cervical vertebra; and (iii) a head immobilization device, which is a nylon skeleton with 30 degrees of horizontal rotation that enables exposure of the visual field during operation. The apparatus can be linked to the operating table and does not contain any metal components that could interfere with the X‐ray penetration in the upper and lower directions of the neck. A patent (Patent No. CN201511031449.5) was obtained for the apparatus.

**Fig. 3:**
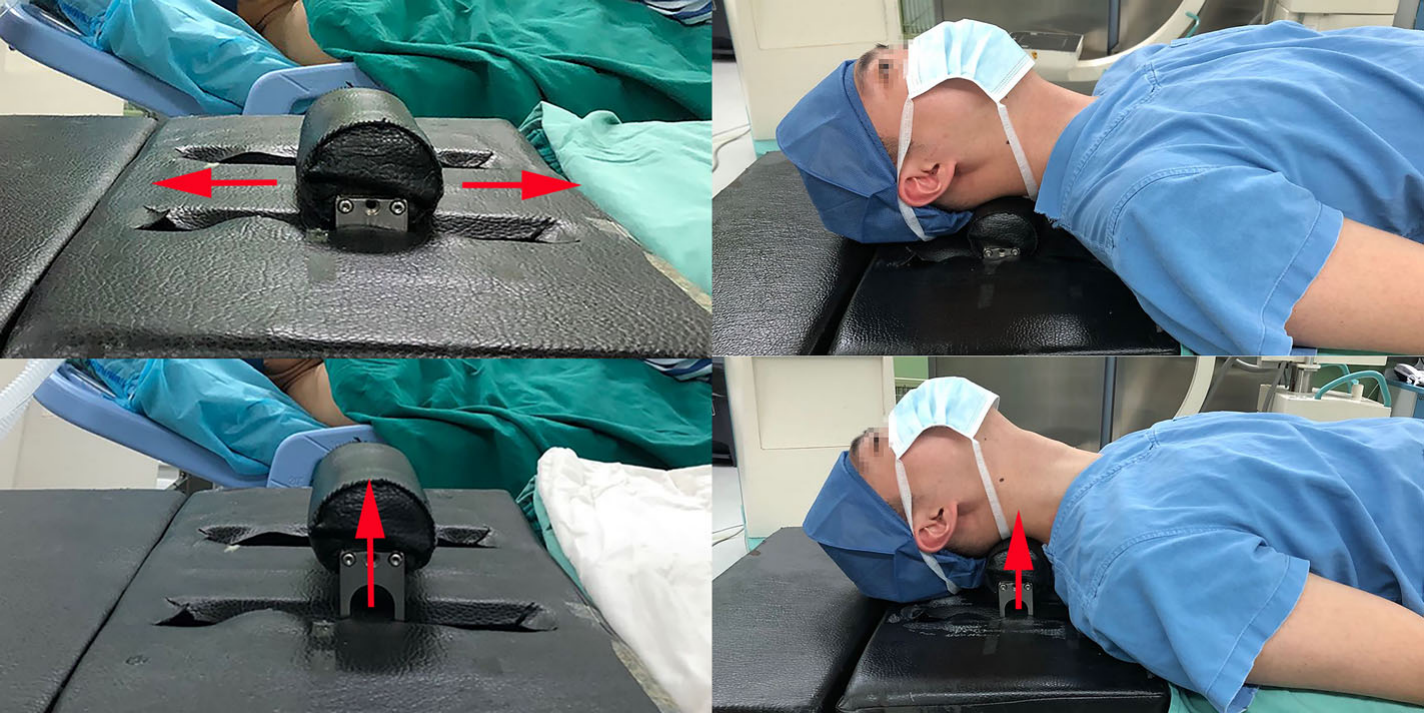
The effect of intraoperative posture‐adjustment apparatus (IPAA). With the cervical electric pillow arising, the neck is altered into extended position.

Following the induction of anesthesia, the IPAA was positioned beneath the neck with a movable pillow placed directly beneath the target levels. The neck was initially placed in a relatively horizontal position to avoid iatrogenic spinal cord or neurological injury. After exposure of the target segment and complete decompression of the spinal canal, we changed the pillow either cranially or caudally or vertically to acquire improved cervical alignment. In addition, we used C‐arm fluoroscopy during adjustment, which can verify the posture of the neck and facet joints so that hyperextension is avoided (Fig. 3).

### Surgical Technique

**Fig. 4:**
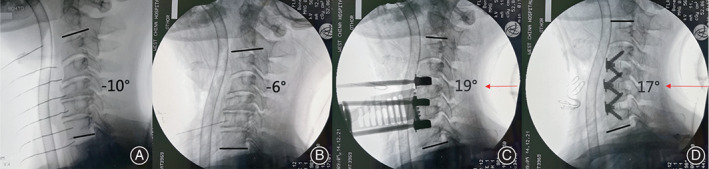
The intraoperative radiograph of a 66‐year‐old woman who underwent anterior cervical discectomy and fusion at C3‐6 level employing the intraoperative posture‐adjustment apparatus. The radiograph showed C2‐C7 lordosis (CL) was −10° at the beginning of the surgery (A). When decompression steps were accomplished, the intraoperative radiograph showed CL was −6° (B). As the pillow arising (labeled with red arrow), the CL changed to 19° (C). When Zero‐P was inserted, the CL suffered a tiny decreased a little to 17° (D).

**Fig. 5:**
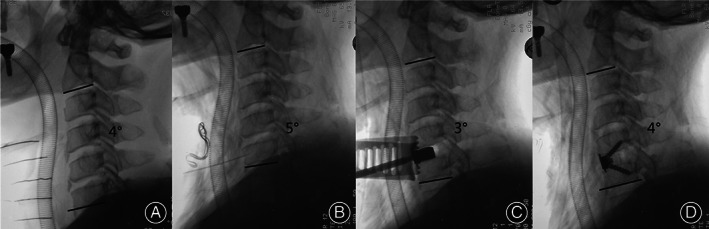
The intraoperative radiograph of a 55‐year‐old man who underwent anterior cervical discectomy and fusion at C5‐6 level employing traditional neck pillow (TNP). The radiograph showed C2‐C7 lordosis (CL) was 4° at the beginning of the surgery (A). When decompression steps were accomplished, the intraoperative radiograph showed CL was 5° (B). For TNP group, the neck posture was settled before surgery and remained unchanged (C). When Zero‐P was inserted, the CL suffered a tiny remained at 4° (D).

All procedures were performed by one surgeon at our hospital. The patients were placed in the supine position after receiving general anesthesia, and the right Smith–Robinson technique was used to expose the surgical target levels. Several steps were required to be undertaken to achieve complete decompression. The posterior longitudinal ligament was completely resected after the ruptured intervertebral disc and osteophytes were removed. Endplate preparation was an important step that should not be neglected when promoting implant insertion. When all decompression steps were completed, the movable pillow must be adjusted to achieve better cervical alignment. No additional compression was imposed on the spinal cord as all decompression materials were removed when the neck position was set (Fig. 4). There were no such adjustment steps in the TNP group as the neck position settled at the beginning of the surgery and remained unchanged throughout (Fig. 5). Finally, an appropriately sized implant was selected and inserted into the intervertebral space based on the height and width of the intervertebral disc.

### Radiologic Evaluation

Radiological parameters were recorded pre‐ and post‐operatively and at the 3‐month, 6‐month, and 1‐year follow‐ups for each patient. In this study, we innovatively recorded and measured intraoperative x‐rays to directly observe the effect of the IPAA in adjusting cervical alignment. Radiological parameters such as the degree of C2‐C7 lordosis (CL), functional spinal unit angle (FSUA), C7 slope (C7S), and fusion rate were recorded. C7 was more visible than T1 in most patients, and we therefore measured the levels of C7S instead of T1S. CL is defined as the angle between the lower endplates of C2 and C7, and FSUA is the segmental angle, which was measured by drawing two lines of the upper and lower endplates of the target level. C7S is the angle formed between the horizontal plane and the C7 upper endplate. Fusion was evaluated using X‐rays in combination with CT. Bony fusion was defined as: (i) no more than 2° of angular motion on dynamic radiographs; (ii) the absence of a radiolucent gap between the grafts and endplates; and (iii) the presence of continuous bridging bony trabeculae at the graft‐endplate interface. ASD was defined as at least one of the following findings on radiography: calcification of the anterior longitudinal ligament, narrowing of the disc space with or without posterior osteophytes, or new anterior or enlarged osteophyte formation.^19^

### Clinical Evaluation

The Japanese Orthopaedic Association (JOA) score, arm and neck visual analogue scale (VAS) score, and neck disability index (NDI) were used to evaluate the clinical outcomes at the pre‐operative, immediately post‐operative, and final follow‐up stages. The Braden Scale was used to evaluate the risk of pressure ulcers caused by the intraoperative position at post‐operative follow‐up.^20^ Dysphagia was assessed by using the Bazaz system.^21^ Other complications including kyphosis, revision surgery, hematoma, cerebrospinal fluid leakage, wound infection, and deep venous thrombosis were also recorded.

### Statistical Analysis

Statistical analysis was conducted using SPSS software (version 25.0, 2018; IBM Corp., Armonk, NY, USA). The results are presented as the means ± standard deviation (SD) when the data satisfied the criteria for normality. Otherwise, the results are presented as the median (range). The independent *t*‐test or Mann–Whitney *U* test was used to compare continuous data between the two groups. Categorical variables were assessed using the Pearson's chi‐square test or Fisher's exact test, and a paired *t*‐test was used to analyze the differences between pre‐operative and post‐operative parameters. Univariate linear regression analysis was used to analyze the association between pillow‐rising distance and changes in CL values. Statistical significance was set at *p* < 0.05.

## Results

### Intraoperative Radiological Outcomes

**Fig. 6:**
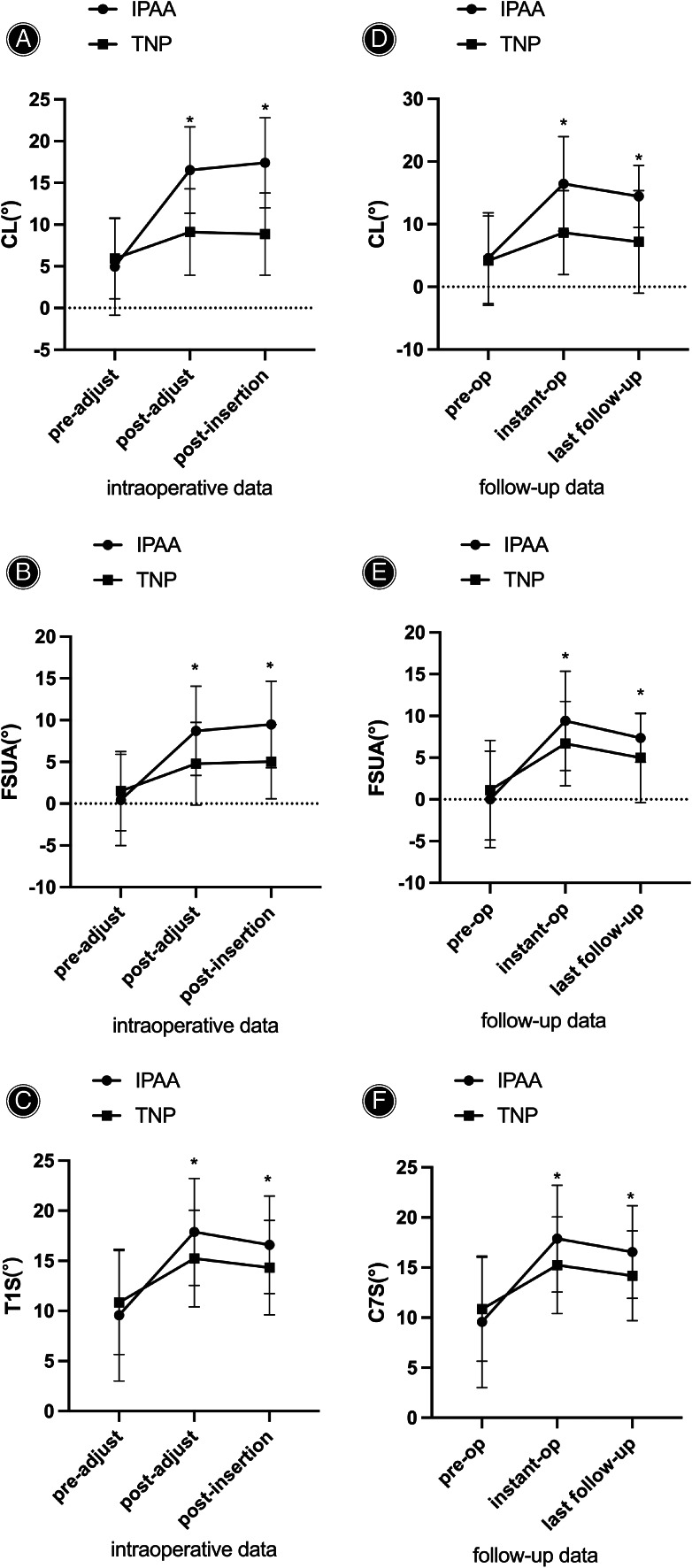
Comparison of C2‐C7 lordosis (CL), functional spinal unit angle (FSUA), C7 slope (C7S) in intraoperative posture‐adjustment apparatus (IPAA) group and traditional neck pillow (TNP) group. Results are expressed as mean ± SD. A dragger represents a significant difference between two groups. Through intraoperative radiograph and follow‐up radiograph, CL, FSUA, C7S in the IPAA group were significantly higher than that in the TNP group.

According to the intraoperative x‐ray, CL in the IPAA group increased from pre‐adjustment 4.97 ± 5.84° to post‐adjustment 16.54 ± 5.18°, and it increased slightly and remained at 17.41 ± 5.41° following implant insertion, whereas CL in the TNP group increased from pre‐adjustment 5.93 ± 4.82° to post‐adjustment 9.11 ± 5.17° and suffered a slight decrease to 8.86 ± 4.92°. As for FSUA, it increased in the IPAA group from pre‐adjustment 0.45 ± 5.46° to post‐adjustment 8.72 ± 5.34° and increased slightly and maintained at 9.49 ± 5.18° following implant insertion, while FSUA in the TNP group increased from pre‐adjustment 1.50 ± 4.73° to post‐adjustment 4.79 ± 4.94° and increased slightly and maintained at 5.04 ± 4.45°. C7S in the IPAA group grew from pre‐adjustment 9.58 ± 6.57° to post‐adjustment 17.89 ± 5.33° and decreased slightly to 16.60 ± 4.86° after implant insertion, while C7S in the TNP group climbed from pre‐adjustment 10.84 ± 5.19° to post‐adjustment 15.23 ± 4.83° and decreased to 14.33 ± 4.70° after implant insertion. The pre‐adjustment differences in CL, FSUA, and C7S between the IPAA and TNP groups were not statistically significant (*p* > 0.05). However, post‐adjustment and post‐insertion differences in CL, FSUA, and C7S were significantly different between the two groups (16.54 ± 5.18° *vs*. 9.11 ± 5.17°, 8.72 ± 5.34° *vs*. 4.79 ± 4.94°, 17.89 ± 5.33° *vs*. 15.23 ± 4.83°; *p* < 0.05) (Fig. 6A–C).

### Follow‐up Radiological Outcomes

CL in the IPAA group increased from 4.57 ± 7.26° preoperatively to 16.45 ± 7.54° immediately postoperatively and decreased to 14.44 ± 4.94° at the last follow‐up, while CL in the TNP group increased from 4.18 ± 7.13° preoperatively to 8.65 ± 6.69° immediately post‐operatively and suffered a slight decrease to 7.17 ± 8.19° at the last follow‐up. In terms of FSUA, in the IPAA group, it increased from 0.01 ± 5.77° preoperatively to 9.41 ± 5.96° immediately post‐operatively and decreased and maintained at 7.36 ± 2.91° at the last follow‐up, whereas FSUA in the TNP group increased from 1.11 ± 5.96° preoperatively to 6.68 ± 5.05° immediately post‐operatively and decreased majorly and maintained at 4.99 ± 5.36° at the last follow‐up. C7S in the IPAA group increased from 9.57 ± 6.57° preoperatively to 17.89 ± 5.33° immediately post‐operatively and decreased and maintained at 16.54 ± 4.63° at the last follow‐up, while C7S in the TNP group increased from 10.85 ± 5.18° preoperatively to 15.23 ± 4.83° immediately post‐operatively and decreased minorly and maintained at 14.19 ± 4.48° at the last follow‐up (Fig. 6E–G).

**Fig. 7:**
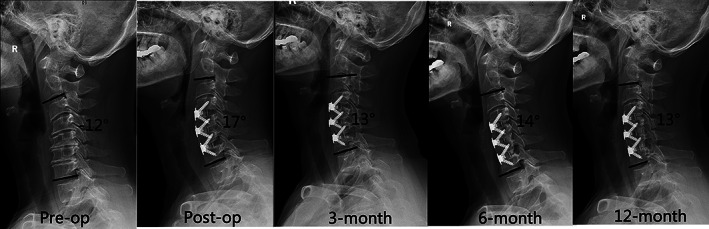
The follow‐up radiograph of patient employing intraoperative posture‐adjustment apparatus (IPAA). C7 slope (CL) increased from pre‐operative −12° to post‐operative 17°. Although the CL suffered a little decrease to 13° at 3‐month follow‐up, correction of CL was well maintained at 12‐month follow‐up.

**Fig. 8:**
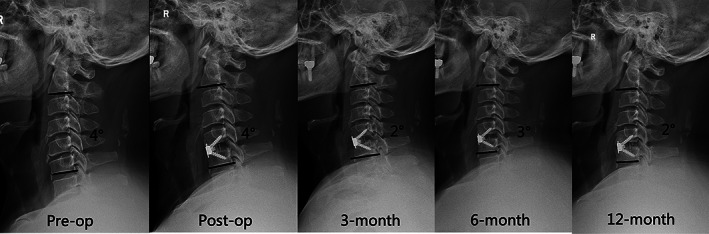
The follow‐up radiograph of patient employing traditional neck pillow (TNP). C7 slope (CL) was almost unchanged from pre‐operative 4° to post‐operative 4° and decreased to 2° at 12‐month follow‐up.

Pre‐operative cervical alignment parameters such as CL, FSUA, and C7S were not significantly different between the two groups (*p* > 0.05). Compared with the pre‐operative data, CL, FSUA, and C7S increased significantly in both groups immediately post‐operatively and were maintained at the last follow‐up (*p* < 0.05). The IPAA group had significantly greater CL, FSUA, and C7S than the TNP group (16.45 ± 7.54° *vs*. 8.65 ± 6.69°, 9.41 ± 5.96° *vs*. 6.68 ± 5.05, and 17.89 ± 5.33*° vs*. 15.23 ± 4.83°; *p* < 0.05). Additionally, such differences between the two groups persisted significantly even at the last follow‐up (14.44 ± 4.94° *vs*. 7.17 ± 8.19°, 7.36 ± 2.91° *vs*. 4.99 ± 5.36°, and 16.54 ± 4.63° *vs*. 14.19 ± 4.48°; *p* < 0.05). (Figs. 7 and 8).

In addition, we measured the arising distance of the neck pillow to evaluate whether there was any correlation between the arising distance and the changes in cervical alignment. Univariate linear regression analysis showed that the pillow rising distance was positively associated with changes in CL values (*β* = 3.199, *p* < 0.001).

At the last follow‐up, the fusion rate in the IPAA group was 95% (55/58), whereas in the TNP group it was 93% (26/28); this rate was not significantly different between the two groups (*p* > 0.05). Kyphosis was observed in two patients in the TNP group but not in the IPAA group (*p* = 0.041) (Table 3), and no ASD was observed in either group at the final follow‐up (Table 3).

### Clinical Outcomes

Most patients experienced relief from their symptoms after surgery. Compared to the pre‐operative parameters, JOA scores increased significantly post‐operatively (*p* < 0.05). The neck and arm VAS and NDI scores decreased significantly post‐operatively compared to the pre‐operative baseline measurements (Table 2) (*p* < 0.05). The NDI and VAS neck scores in the IPAA group were significantly lower post‐operatively and at the last follow‐up than those in the TNP group (*p* < 0.05), although there was no significant difference in the VAS arm and JOA scores between the two groups (*p* > 0.05).

**TABLE 2:** Clinical outcomes (displayed as mean ± standard deviation)

	IPAA group (*n* = 58)	TNP group (*n* = 28)	*t/x* ^*2*^ */z*	*p*
JOA				
Pre‐op	9.00 ± 2.89	9.07 ± 2.57	0.217	0.89
Post‐op	13.40 ± 2.19	13.61 ± 2.25	−0.605	0.63
Last follow‐up	14.33 ± 2.10	14.57 ± 2.20	0.783	0.58
NDI				
Pre‐op	26.52 ± 2.49	26.77 ± 2.39	−1.327	0.61
Post‐op	14.04 ± 2.10	16.34 ± 2.15	−0.424	<0.001
Last follow‐up	6.90 ± 1.36	9.88 ± 2.12	−1.870	<0.001
VAS neck				
Pre‐op	6.44 ± 1.30	6.29 ± 1.26	1.202	0.55
Post‐op	2.20 ± 1.27	3.25 ± 1.14	0.292	<0.001
Last follow‐up	1.29 ± 1.12	2.17 ± 1.04	1.442	<0.001
VAS arm				
Pre‐op	6.04 ± 1.65	6.45 ± 1.36	1.303	0.17
Post‐op	3.06 ± 1.34	3.46 ± 1.55	0.389	0.16
Last follow‐up	1.44 ± 0.62	1.66 ± 0.98	1.224	0.18

Abbreviations: IPAA, intraoperative posture‐adjustment apparatus; JOA, Japanese Orthopaedic Association; NDI, neck disability index; TNP, traditional neck pillow; VAS, visual analog scale.

### Complications

According to the Braden Scale, two patients in the IPAA group and one patient in the TNP group had a low risk of developing pressure ulcers, with no significant difference between the two groups (*p* = 0.98, Table 3). Dysphagia was observed in only one patient in the IPAA group at the last follow‐up. No post‐operative cerebrospinal fluid leakage, revision surgery, hematoma wound infection, or deep venous thrombosis were observed.

**TABLE 3:** Comparison fusion rate and complications in IPAA and TNP groups

Variable	IPAA group (*n* = 58)	TNP group (*n* = 28)	*t/x* ^*2*^ */z*	*p*
Fusion rate	55 (95%)	26 (93%)	0.134	0.714
ASD	0	0		‐
Braden scale classification			0.585	0.977
Elevated risk (10 to 12)	0	0		
Moderate risk (13 to 14)	0	0		
At risk (15 to 18)	2	1		
No risk (19 or higher)	56	27		
Dysphagia	1	0	0.488	0.485
Pre‐operative Kyphosis	11	3	0.943	0.331
Last follow‐up kyphosis	0	2	4.241	0.039

Abbreviations: ASD, adjacent segment disease; IPAA, intraoperative posture‐adjustment apparatus; TNP, traditional neck pillow.

## Discussion

### Main Findings

Our results showed that IPAA was better at adjusting for CL, FSUA, and C7S than the TNP group (*p* < 0.05). Additionally, the NDI and VAS neck scores in the IPAA group were significantly lower than those in the TNP group (*p* < 0.05). At the last follow‐up, the TNP group had significantly more kyphotic patients than the IPAA group (2 *vs*. 0, *p* = 0.041). There was no significant difference between the two groups in terms of fusion rates, ASD incidence, or complications such as dysphagia, Braden's scale score, revision surgery, hematoma, cerebrospinal fluid leakage, wound infection, and deep venous thrombosis.

### IPAA's Role in Adjusting Cervical Alignment and Avoiding Spinal Cord Injury

Cervical spinal alignment following cervical surgery has gained considerable interest over the last decade.^22^, ^23^, ^24^ However, the sagittal correction standards for cervical spine surgery have not always been consistent. Suda *et al*. reported kyphosis to be associated with less favorable clinical outcomes,^25^, ^26^ and Yoshida *et al*. concluded that anterior cervical surgery with lordosis reconstruction is an appropriate option, especially for patients with kyphosis.^27^ Some researchers have also contended that an appropriate cervical spine lordosis alignment may lower the incidence of ASD.^28^, ^29^, ^30^

However, multiple studies have reported intraoperative spinal cord injuries. According to Hindman *et al*., cervical cord injury claims account for 48 out of 5231 (0.9%) claims for general anesthesia procedures.^11^ Additionally, studies by Flynn and by Lee have reported incidence rates of postoperative spinal cord injury of 0.3% and 0.1%, respectively.^31^, ^32^ Schwartz *et al*. retrospectively reviewed 3806 patients who underwent anterior cervical spine surgery and found that 69 (1.8%) patients had neurological injuries.^33^ All of these studies demonstrated that iatrogenic spinal cord injuries result from improper neck positioning and are seemingly inevitable, particularly in cervical spine surgery.

To help patients achieve better cervical alignment, we opted to place the neck in a slightly extended position. However, for patients with severe compression of the spinal cord or cervical instability, we preferred to avoid this extended posture as it could aggravate spinal cord compression. Consequently, such patients may not achieve satisfactory cervical lordosis. Therefore, avoiding iatrogenic spinal cord injury while simultaneously assisting patients in achieving satisfactory cervical alignment remains an ambiguous topic in cervical surgery. Thus, how to strike a balance between effectiveness and safety in adjusting cervical alignment arises as an essential question.

Traditionally, TNP has been used to restore cervical lordosis following anesthesia. Notably, the extended position sustained by TNP is fixed and unchangeable intraoperatively. One of the problems with TNP is poor adjustment in patients with severe spinal cord compression or cervical instability. To overcome these shortcomings, we developed an innovative IPAA. With the IPAA, we could manipulate the neck in an extended position after anterior cervical decompression. By switching the order of the neck position setting and decompression steps, iatrogenic spinal cord injury caused by an inappropriate neck position could be avoided. This is obviously true, as when the decompression steps are achieved, there are no herniated disc or osteophytes that could compress the spinal cord even with an extremely extended neck position. This theory has been exemplified in our study, which showed that IPAA had significantly better clinical and radiological outcomes than those of TNP.

### Analysis of IPAA Efficiency

The intraoperative data showed that the adjustment of the cervical alignment in the IPAA group was significantly superior to that in the TNP group (*p* < 0.05). Univariate linear regression analysis showed that the pillow rising distance was positively correlated with changes in the CL values (*β* = 3.199, *p* < 0.001). These results illustrate that the IPAA enhances the effectiveness of cervical adjustment. The follow‐up data showed that adjustment of the cervical alignment in the IPAA group was well maintained at the last follow‐up; however, two patients in the TNP group remained kyphotic after anterior cervical surgery. The IPAA group had significantly lower NDI and VAS neck scores than the TNP group (*p* < 0.05), indicating that IPAA can provide patients with satisfactory clinical outcomes. The findings are consistent with those reported by Lee *et al*.: CL in lordotic curve traction group increased from 4.8 ± 10.9° to 16.9 ± 12.7° while that in traditional traction group remained unchanged around 5°.^24^ Cao *et al*. have reported that mild neck pain group owned a significantly higher pre‐operative and post‐operative CL than that in severe neck pain group (11.88 ± 7.41° *vs*. 6.33 ± 6.53°, 16.64 ± 7.34° *vs*. 13.49 ± 5.31; *p* < 0.05).^34^ Recent studies have found that CL values vary widely and increase gradually with increasing age. At present, the sagittal correction for cervical spine surgery has not been uniform. As for the fusion rate and complication rates such as the Braden scale score, dysphagia, and cerebrospinal fluid leakage, no significant differences were noted, implying adequate safety for this IPAA apparatus (*p* > 0.05).

### Relationship between Cervical Sagittal Alignment Balance Index and Postoperative Clinical Outcomes

Direct decompression, stability of fused segments, and most importantly, restoration of cervical lordosis are prerequisites for successful ACDF. Multiple studies have demonstrated the relationship between cervical sagittal balance and neck pain, cervical disc degeneration, and ACDF prognosis.^22^, ^23^, ^24^, ^25^, ^26^ CL and FSUA are conventional parameters used to measure cervical alignment. Previous studies have indicated that these parameters strongly correlate with patient outcomes after anterior cervical surgery, particularly ACDF. Cao *et al*. reported that the incidence of post‐operative axial pain was 33%, and logistic regression demonstrated that the pre‐operative C2–C7 Cobb angle was an independent predictor of post‐operative axial pain.^34^ Iyer *et al*. enrolled 90 patients and reported the average CL to be 13.7 ± 14.9°, showing that increasing CL and T1S are correlated with decreasing NDI.^35^ The results of our research suggested that the IPAA group had better cervical alignment and smaller VAS and NDI scores than the TNP group. In light of the aforementioned studies, sustaining proper cervical alignment is crucial when performing anterior cervical surgery. T1S serves as the foundation of the cervical spine and is an important parameter. The orientation of the T1 vertebra is one of the determinants of global sagittal balance. Jouibari *et al*. concluded that T1S was significantly different in the neck pain (27.7 ± 6.29°) and pain‐free groups (32.5 ± 8.0°).^36^ Several authors have recommended that C7 is clearer and more visible than T1.^37^ In this study, we measured C7S instead of T1S owing to its easy visibility. In accordance with previous research, we demonstrated that the C7S in the IPAA group was superior to that in the TNP group. Another study by Lee *et al*. enrolled 40 patients and observed that lordotic curve‐controlled traction is better than traditional horizontal traction in CL (16.9 ± 12.7° vs. 4.9 ± 9.8°) and clinical outcomes.^38^

### Innovative and Creative Features of IPAA

The Jackson table was designed specifically for spinal surgery and the neck position could not be changed during surgery, and a novel positioning system for posterior cervical surgery has been previously described in prior studies.^39^ However, a posture adjustment apparatus for anterior cervical surgery has not yet been reported. In a double‐blind randomized controlled trial, Lee *et al*. compared the clinical and radiological outcomes of cervical lordotic curve‐controlled traction with those of conventional traction. Their research demonstrated that both clinical and radiological outcomes of lordotic curve‐controlled traction were better than those of conventional traction.^38^ Thus, to achieve postural adjustment during anterior cervical surgery, we invented and created an IPAA. Our study is the first to describe an intraoperative positioning adjustment strategy specifically for anterior cervical surgery, which showed better radiological and clinical outcomes with IPAA than with TNP. This could be regarded as a supplement to the Jackson table and would make the intraoperative positioning setting realistic. With the use of this novel device, patients with a positive neck extension test, severe kyphosis, or myelopathy accompanying spinal stenosis may benefit from better‐aligned cervical spines.

### Optimal IPAA Utilization

To use IPAA at its fullest potential, several methods have been noted. First, despite the fact that this pillow can move cranially and caudally, it is preferable to set the pillow directly beneath the target segment or where sagittal malalignment occurs before administering anesthesia. This offers efficiency regarding time and precision in cervical angle correction. Second, intraoperative extension position changes should strictly follow a thorough and complete decompression. Once the decompression factors are removed, there is no threat to the spinal cord even when the neck is in an extremely extended position. This is important as it ensures the safety of the IPAA. Third, a Caspar distractor can be used as a supplementary way to achieve intraoperative cervical alignment adjustment. However, the Caspar distractor plays a limited role in cervical alignment correction, particularly in patients with osteoporosis.^40^, ^41^ Fourth, although there is still no precise target angle that should be corrected, fluoroscopy should be employed to avoid over‐distraction of the facet joint and anterior intervertebral disc height, as doing so would lead to posterior neck pain and disability.^34^, ^42^, ^43^ According to Bai *et al*., an intervertebral height change of >10% leads to post‐operative axial pain. Further observations should be performed to deduce the appropriate extent of distraction.^44^ Finally, after insertion of the implant, the pillow should be slightly manipulated downward to prevent high pressure, which may cause pressure ulcers at the contact surface between the neck skin and the pillow.

### Strengths and Limitations

To the best of our knowledge, this is the first study to investigate and apply an IPAA in anterior cervical surgery. The IPAA makes it possible to properly correct cervical alignment and guarantee the safety of anterior cervical surgery, especially in patients with severe spinal cord compression. This study is the first to evaluate and document intraoperative radiographic cervical alignment, clarifies how cervical alignment is corrected *via* IPAA, and highlights the importance of this device. However, our study has some limitations. First, due to the short follow‐up period, it was impossible to draw any conclusions on the long‐term effects of this new posture‐adjusting apparatus such as ASD. Additionally, a larger sample size of patients using this apparatus should be enrolled in future studies, as our sample size was quite modest. Furthermore, although this device exemplified improved cervical lordosis, future studies should also define and explain optimal lordosis.

## Conclusion

The IPAA achieves intraoperative postural adjustment during anterior cervical surgery. IPAA may offer convenience for surgeons in rebuilding cervical lordosis, as it can be used to change the neck position while simultaneously avoiding iatrogenic spinal cord injury. Posture adjustment is a safe and effective method for changing neck position during surgery and obtaining better cervical sagittal alignment; furthermore, patients with IPAA had better clinical outcomes in terms of the NDI and neck VAS scores in this study. We believe that this innovative device is a reasonable alternative for patients with a positive neck extension test, cervical kyphosis, or severe spinal stenosis to achieve satisfactory cervical lordosis and avoid spinal cord injury.

## Funding Information

This study was supported by the Sichuan Province Science and Technology Support Program of China (Grant No. 2019YFQ0002 to H Liu, Grant No. 2020YFS0084 to B Wang), West China Nursing Discipline Development Special Fund Project, Sichuan University (Grant No. HXHL19016 to Y Hong), Science And Technology Project of The Health Planning Committee of Sichuan (Grant No. 21PJ039 to T Wu).

## Conflict of Interest

The authors declare they have no competing interests.

## Ethical Statement

This retrospective, clinical, and comparative study was approved by the Ethics Committee of the West China Hospital (No. 2020–744). All the experiments were performed in accordance with the Declaration of Helsinki.

## Ethics Approval and Consent to Participate

This study approved by the Institutional Review Board of West China Hospital. Informed consent was obtained from all individual participants included in this study.

## Author Contributions

Yifei Deng and Beiyu Wang wrote the paper and contributed equally to this work and should be considered as co‐first authors. Ying Hong and Hao Liu take responsibility for the integrity of the work as a whole from inception to published article and should be designated as corresponding authors. Hao Liu, Yifei Deng, Yang Meng, Beiyu Wang, Ying Hong contributed to the concept and design of this study. Yifei Deng, Ying Hong, Yang Meng, Xin Rong, Tingkui Wu, Hao Chen contributed to literature search, data extraction, bias assessment and data analysis. Ying Hong, Hao Liu contributed to manuscript preparation, editing and review.

## Consent for Publication

Informed consent was obtained from all individual participants included in this study.

## Declaration of Approval

All the authors have read and approved this manuscript.

## Data Availability

Datasets are available from the corresponding author on reasonable request.

## References

[1] Kim SW , Limson MA , Kim SB , Arbatin JJF , Chang KY , Park MS , et al. Comparison of radiographic changes after ACDF versus Bryan disc arthroplasty in single and bi‐level cases. Eur Spine J. 2009;18:218–231. 10.1007/s00586-008-0854-z 19127374 PMC2899339

[2] Fraser JF , Hartl R . Anterior approaches to fusion of the cervical spine: a meta‐analysis of fusion rates. J Neurosurg Spine. 2007;6:298–303. 10.3171/spi.2007.6.4.2 17436916

[3] Burkhardt BW , Brielmaier M , Schwerdtfeger K , Sharif S , Oertel JM . Smith‐Robinson procedure with an autologous iliac crest graft and Caspar plating: report of 65 patients with an average follow‐up of 22 years. World Neurosurg. 2016;90:244–250. 26945983 10.1016/j.wneu.2016.02.074

[4] Burkhardt BW , Brielmaier M , Schwerdtfeger K , Oertel JM . Clinical outcome following anterior cervical discectomy and fusion with and without anterior cervical plating for the treatment of cervical disc herniation‐a 25‐year follow‐up study. Neurosurg Rev. 2018;41:473–482. 28646343 10.1007/s10143-017-0872-6

[5] Phillips FM , Geisler FH , Gilder KM , Reah C , Howell KM , McAfee PC . Long‐term outcomes of the US FDA IDE prospective, randomized controlled clinical trial comparing PCM cervical disc arthroplasty with anterior cervical discectomy and fusion. Spine (Phila Pa 1976). 2015;40(10):674–683. 25955086 10.1097/BRS.0000000000000869

[6] Gornet M , Burkus J , Shaffrey M , Schranck F , Copay A . Cervical disc arthroplasty: 10‐year outcomes of the prestige LP cervical disc at a single level. J Neurosurg Spine. 2019;31:1–9. 31075769 10.3171/2019.2.SPINE1956

[7] Radcliff K , Davis RJ , Hisey MS , Nunley PD , Hoffman GA , Jackson RJ , et al. Long‐term evaluation of cervical disc arthroplasty with the Mobi‐C© cervical disc: a randomized, prospective, multicenter clinical trial with seven‐year follow‐up. Int J Spine Surg. 2017;11(4):31. 29372135 10.14444/4031 PMC5779239

[8] Shin DA , Yi S , Yoon DH , Kim KN , Shin HC . Artificial disc replacement combined with fusion versus two‐level fusion in cervical two‐level disc disease. Spine (Phila Pa 1976). 2009;34(11):1153–1159. 19444062 10.1097/BRS.0b013e31819c9d39

[9] Wang K‐F , Duan S , Zhu Z‐Q , Liu H‐Y , Liu C‐J , Xu S . Clinical and radiologic features of 3 reconstructive procedures for the surgical Management of Patients with bilevel cervical degenerative disc disease at a minimum follow‐up period of 5 years: a comparative study. World Neurosurg. 2018;113:e70–e76. 29408574 10.1016/j.wneu.2018.01.157

[10] Grasso G . Clinical and radiological features of hybrid surgery in multilevel cervical degenerative disc disease. Eur Spine J. 2015;24(7):842–848. 26463866 10.1007/s00586-015-4281-7

[11] Hindman BJ , Palecek JP , Posner KL , Domino KB . Cervical spinal cord, root, and bony spine injuries: a closed claims analysis. Anesthesiology. 2011;114(4):782–795. 10.1097/ALN.0b013e3182104859 21326090

[12] Rao R . Neck pain, cervical radiculopathy, and cervical myelopathy: pathophysiology, natural history, and clinical evaluation. J Bone Joint Surg Am. 2002;84:1872–1881. 12377921 10.2106/00004623-200210000-00021

[13] Baron EM , Young WF . Cervical spondylotic myelopathy: a brief review of its pathophysiology, clinical course, and diagnosis. Neurosurgery. 2007;60(1 suppl 1):S35–S41. 17204884 10.1227/01.NEU.0000215383.64386.82

[14] Shedid D , Benzel EC . Cervical spondylosis anatomy: pathophysiology and biomechanics. Neurosurgery. 2007;60(1 suppl 1):S7–S13. 17204889 10.1227/01.NEU.0000215430.86569.C4

[15] Muhle C , Metzner J , Weinert D , Falliner A , Brinkmann G , Mehdorn MH , et al. Classification system based on kinematic MR imaging in cervical spondylitic myelopathy. Am J Neuroradiol. 1998;19:1763–1771. 9802503 PMC8337474

[16] Muhle C , Weinert D , Falliner A , Wiskirchen J , Metzner J , Baumer M , et al. Dynamic changes of the spinal canal in patients with cervical spondylosis at flexion and extension using magnetic resonance imaging. Invest Radiol. 1998;33:444–449. 9704283 10.1097/00004424-199808000-00004

[17] Chen CJ , Hsu HL , Niu CC , Wang LJ , et al. Cervical degenerative disease at flexion‐extension MR imaging: prediction criteria. Radiology. 2003;227(1):136–142. 10.1148/radiol.2271020116 12601192

[18] Farmer JC , Wisneski RJ . Cervical spine nerve root compression. An analysis of neuroforaminal pressures with varying head and arm positions. Spine. 1994;19:1850–1855. 7973984 10.1097/00007632-199408150-00010

[19] Lee SE , Jahng TA , Kim HJ . Correlation between cervical lordosis and adjacent segment pathology after anterior cervical spinal surgery. Eur Spine J. 2015;24:2899–2909. 10.1007/s00586-015-4132-6 26198705

[20] Oliveira HMBS , Santos AMJFD , Madeira MZA , et al. Risk assessment for the development of perioperative lesions due to surgical positioning. Rev Gaucha Enferm. 2019;40:e20180114. 10.1590/1983-1447.2019.20180114 31859708

[21] Huang C , Abudouaini H , Wang B , Liu H , et al. Comparison of patient‐reported postoperative dysphagia in patients undergoing one‐level versus two‐level anterior cervical discectomy and fusion with the zero‐P implant system. Dysphagia. 2021;36(4):743–753. 10.1007/s00455-020-10197-w 33387002

[22] Oitment C , Watson T , Lam V , Aref M , Koziarz A , Kachur E , et al. The role of anterior cervical discectomy and fusion on relieving axial neck pain in patients with single‐level disease: a systematic review and meta‐analysis. Global Spine J. 2020;10(3):312–323. 32313797 10.1177/2192568219837923 PMC7160803

[23] Kuntz C , Levin LS , Ondra SL , Shaffrey CI , Morgan CJ . Neutral upright sagittal spinal alignment from the occiput to the pelvis in asymptomatic adults: a review and resynthesis of the literature. J Neurosurg Spine. 2007;6:104–112. 17330576 10.3171/spi.2007.6.2.104

[24] Lee SH , Hyun SJ , Jain A . Cervical sagittal alignment: literature review and future directions. Neurospine. 2020;17(3):478–496. 10.14245/ns.2040392.196 33022153 PMC7538362

[25] Sakai K , Yoshii T , Hirai T , Arai Y , Shinomiya K , Okawa A . Impact of the surgical treatment for degenerative cervical myelopathy on the preoperative cervical sagittal balance: a review of prospective comparative cohort between anterior decompression with fusion and laminoplasty. Eur Spine J. 2017;26:104–112. 27473211 10.1007/s00586-016-4717-8

[26] Suda K , Abumi K , Ito M , Shono Y , Kaneda K , Fujiya M . Local kyphosis reduces surgical outcomes of expansive open‐door laminoplasty for cervical spondylotic myelopathy. Spine. 2003;12:1258–1262. 10.1097/01.BRS.0000065487.82469.D9 12811268

[27] Yoshida G , Alzakri A , Pointillart V , Boissiere L , Obeid I , Matsuyama Y , et al. Global spinal alignment in patients with cervical spondylotic myelopathy. Spine. 2018;43:E154–E162. 10.1097/BRS.0000000000002253 28542100

[28] Hansen MA , Dip G , Kim HJ , et al. Does postsurgical cervical deformity affect the risk of cervical adjacent segment pathology? Spine. 2012;37:S75–S84. 22885832 10.1097/BRS.0b013e31826d62a6

[29] Snowden R , Miller J , Saldon T , et al. Does index level sagittal alignment determine adjacent level disc height loss? J Neurosurg Spine. 2019;31:579–586. 10.3171/2019.4.SPINE181468 31226683

[30] Park MS , Kelly MP , Lee DH , Min WK , Rahman R'KK , Riew KD . Sagittal alignment as a predictor of clinical adjacent segment pathology require surgery after anterior cervical arthrodesis. Spine J. 2014;14:1228–1234. 24361126 10.1016/j.spinee.2013.09.043 PMC4019713

[31] Flynn TB . Neurologic complications of anterior cervical interbody fusion. Spine. 1982;7:536–539. 7167824 10.1097/00007632-198211000-00004

[32] Lee JY , Hilibrand AS , Lim MR , Zavatsky J , Zeiller S , Schwartz DM , et al. Characterization of neurophysiologic alerts during anterior cervical spine surgery. Spine. 2006;31:1916–1922. 10.1097/01.brs.0000228724.01795.a2 16924208

[33] Schwartz DM , Sestokas AK , Hilibrand AS , Albert TJ , et al. Neurophysiological identification of position‐induced neurologic injury during anterior cervical spine surgery. J Clin Monit Comput. 2006;20:437–444. 10.1007/s10877-006-9032-1 16960753

[34] Cao Y , Xu C , Sun B , Liu Y , et al. Preoperative cervical cobb angle is a risk factor for postoperative axial neck pain after anterior cervical discectomy and fusion with zero‐profile interbody. Orthop Surg. 2022;14(12):3225–3232. 10.1111/os.13552 36250553 PMC9732633

[35] Iyer S , Nemani VM , Nguyen J , Elysee J , Burapachaisri A , Ames CP , et al. Impact of cervical sagittal alignment parameters on neck disability. Spine (Phila Pa 1976). 2016;41(5):371–377. 26571157 10.1097/BRS.0000000000001221

[36] Jouibari MF , Le Huec JC , Ranjbar Hameghavandi MH , et al. Comparison of cervical sagittal parameters among patients with neck pain and healthy controls: a comparative cross‐sectional study. Eur Spine J. 2019;28(10):2319–2324. 31444609 10.1007/s00586-019-06117-8

[37] Weng C , Wang J , Tuchman A , Wang J , Fu C , Hsieh PC , et al. Influence of T1 slope on the cervical sagittal balance in degenerative cervical spine. Spine (Phila Pa 1976). 2016;41(3):185–190. 26650871 10.1097/BRS.0000000000001353

[38] Lee CH , Heo SJ , Park SH , Jeong HS , Kim SY . The functional and morphological changes of the cervical intervertebral disc after applying lordotic curve controlled traction: a double‐blind randomized controlled study. Int J Environ Res Public Health. 2019;16(12):2162–2168. 31248064 10.3390/ijerph16122162 PMC6617374

[39] Manabe N , Shimizu T , Tanouchi T , Fueki K , Ino M , Toda N , et al. A novel skull clamp positioning system and technique for posterior cervical surgery. Medicine. 2015;94:e695. 10.1097/MD.0000000000000695 25929898 PMC4603043

[40] Wang L , Qiu C , Tian Y , Liu X , et al. Comparative study between Caspar cervical retractor system and traditional S retractor in application on anterior cervical decompression and fixation. Orthop Surg. 2023;15(2):510–516. 10.1111/os.13618 36513624 PMC9891904

[41] Buraimoh M , Basheer A , Taliaferro K , Shaw JH , Haider S , Graziano G , et al. Origins of eponymous instruments in spine surgery. J Neurosurg Spine. 2018;29:696–703. 30215591 10.3171/2018.5.SPINE17981

[42] Kirzner N , Etherington G , Ton L , Chan P , Paul E , Liew S , et al. Relationship between facet joint distraction during anterior cervical discectomy and fusion for trauma and functional outcome. Bone Joint J. 2018;100‐B(9):1201–1207. 10.1302/0301-620X.100B9.BJJ-2018-0199.R1 30168770

[43] Xu C , Wang R , Li J , Zhong H , Zhang Z , Cui C , et al. Intervertebral‐spreader‐assisted anterior cervical discectomy and fusion prevents postoperative axial pain by alleviating facet joint pressure. J Orthop Surg Res. 2022;17(1):91. 35168657 10.1186/s13018-022-02983-z PMC8845354

[44] Bai J , Zhang X , Zhang D , Ding W , Shen Y , Zhang W , et al. Impact of over distraction on occurrence of axial symptom after anterior cervical discectomy and fusion. Int J Clin Exp Med. 2015;8(10):19746–19756. 26770640 PMC4694540

